# Effect of Chronic Administration of Resveratrol on Cognitive Performance during Aging Process in Rats

**DOI:** 10.1155/2017/8510761

**Published:** 2017-10-15

**Authors:** A. R. Navarro-Cruz, R. Ramírez y Ayala, C. Ochoa-Velasco, E. Brambila, R. Avila-Sosa, S. Pérez-Fernández, J. C. Morales-Medina, P. Aguilar-Alonso

**Affiliations:** ^1^Departamento de Bioquímica-Alimentos, Facultad de Ciencias Químicas, Benemérita Universidad Autónoma de Puebla, Puebla, Mexico; ^2^Posgrado en Ciencias Químicas, BUAP, Puebla, Mexico; ^3^Departamento de Análisis Clínicos, Facultad de Ciencias Químicas, Benemérita Universidad Autónoma de Puebla, Puebla, Mexico; ^4^Departamento de Microbiología, Facultad de Ciencias Químicas, Benemérita Universidad Autónoma de Puebla, Puebla, Mexico; ^5^Centro de Investigación en Reproducción Animal, CINVESTAV-Universidad Autónoma de Tlaxcala, Tlaxcala, Mexico

## Abstract

The increase in the elderly population has generated concern to meet health demands. The research efforts to elucidate the mechanisms of damage associated with aging have also been significantly increased, especially in order to avoid the reduction of the cognitive abilities in geriatric patients, resulting from the damage generated mainly at the level of the hippocampus during old age. At present, many studies describe resveratrol as an antiaging component. There are reports that it can activate the Sirt1 gene related to antiaging, emulating the effects obtained by caloric restriction in rodents. The aim of the study was to evaluate the effect of chronic administration of resveratrol (10 mg/kg) on cognitive performance in behavioral tests after 8 months of treatment and on the preservation of cerebral integrity in the cytoarchitecture of regions CA1 and CA2. Results showed that the cytoarchitecture of the CA1 and CA2 regions in the hippocampus retained their integrity over time in rats treated with resveratrol, and the behavioral test performed revealed that chronic resveratrol administration for 8 months showed improvements in cognitive performance. The results indicate that resveratrol may exhibit therapeutic potential for age-related conditions.

## 1. Introduction

One of the most important aspects in the adult stage is the deterioration of functional, emotional, and cognitive capacities. These changes limit the carrying out of the habitual activities necessary for the life of the people with the consequent diminution of their independence and the constant need for help. The aging process has been considered as an inevitable physiological change that occurs in living organisms and that progress over time [1, 2].

There is a growing interest in searching for neuroprotective agents from natural products since they contain compounds with high antioxidant power [3]; in this regard, resveratrol has received considerable attention during the last decade [4]. Resveratrol is a stilbenoid constituted by the union of a phenolic ring and a phytoalexin, naturally produced by 72 different plant species, especially vines, pines, and legumes [5]. The presence of these linked rings confers the antioxidant activity because it has been shown that these structures have the ability to be a scavenger of hydroxyl radicals. The most popular form of consumption is in red wine and nuts [6].

It has been shown that resveratrol can be present in cis/transisoforms, among which only the transisoform is biologically active. It is important to mention that transresveratrol can also be produced biotechnologically as a nutritional supplement from *Polygonum cuspidatum* (*Polygonum*, St. Mary's wort, pejiguera herb, pestle, and partridge paw) [7]. It has been demonstrated that a *Polygonum cuspidatum* extract containing 20% transresveratrol had comprehensive suppressive effects on inflammatory and oxidative stress, decreasing TNF-α levels, interleukin, intranuclear NFκB binding, c-jun-N-terminal kinase 1 (JNK 1), and phosphotyrosine phosphatase-1B (PTP-B), as well as reactive oxygen species (ROS) generation in mononuclear cells [8]. Using the model senescence-accelerated mouse (SAM), it was found that resveratrol extracted from Hu Zhang increased the SOD and GPx activities, while decreasing malondialdehyde (MDA) level in SAM *in vivo*. Resveratrol could improve neuromuscular coordination and sensorimotor ability in tightrope test. It could also enhance the learning and memory capacity in the Morris water maze test in SAM [9].

The bioavailability of resveratrol depends on the food matrix where it is incorporated. In humans, when resveratrol is administered orally, a large number of secondary metabolites are detected in plasma and urine, primarily glucuronides. In addition, the bioavailability of resveratrol depends on the vehicle in which it is immersed, that is, oral doses of grape juice report sulfate in plasma and urine while wine administration has increased evidence of glucuronide [10].

In a lot of bioavailability studies, it has been proposed that resveratrol can prevent some types of cancer [11] and confers neutroprotective properties [12], as well as protective effects in cholestatic liver injury [13]. Resveratrol prolongs the useful life of species such as yeasts, worms, and flies [14] by the activation of the silent regulator of information 2 (SIR2), which belongs to the family of sirtuins and has been frequently related to the increase of longevity in some species. Although the pharmacological actions of resveratrol have been linked to antioxidant activity, the possible link between activation of sirtuins and redox regulation by resveratrol is not yet clear. It should be noted that previously the activity of sirtuins had been reported in organisms subject to caloric restriction, so resveratrol is considered a mimetic of the effects of this nutritional practice [15].

The effects of aging on the brain and cognition are very extensive and have multiple etiologies. As we age, there are changes in macroscopic morphology, increases in blood pressure and, with it, the possibility of stroke. In addition, the brain contracts in volume, particularly in the frontal cortex. This change is determinant because it compromises cognitive functions and has been associated with dementia processes [16]. The mechanism by which the brain volume decreases is not yet clear; however, it has been proposed that it is due to the decrease of gray matter because of neuronal death, white matter loss, and changes in dendritic spines [17]. The consequences that are relevant to this loss of brain function are the cognitive changes associated with all types of memory (semantic, episodic, procedural, and working memory) that lead to behavioral disorders, depression, and dementia processes [16].

In this work, we studied the role of chronic administration of resveratrol in oxidative process, the cytoarchitecture of the hippocampus, and the cognitive processes in different periods of rats' life.

## 2. Materials and Methods

### 2.1. Experimental Animals

3-month-old male Wistar rats were obtained from the Bioterio Claude Bernard of the Benemérita Universidad Autónoma de Puebla. The animals were housed in a controlled temperature and humidity environment conditions with light-dark cycles of 12–12 h, with free access to water and food. All treatment methods used in this study were performed according to the guide for the care and use of laboratory animals NOM-062-ZOO-1999. Every effort was made to minimize the suffering of animals.

### 2.2. Resveratrol Dose Determination

To establish the dose of resveratrol, 30 rats were divided into six groups, according to their administration, divided into the following categories: group administered with vehicle (10% saline and 10% ethanol) and 4 other groups each administered with resveratrol in 2.5, 5, 10, 20, and 50 mg/kg/day, respectively (resveratrol was diluted in physiological solution and 10% ethanol). A stainless steel cannula was used for the oral administration of different doses. Once administrated, rats were returned to their home cage. After daily administration for two months, the rats of diverse groups were sacrificed and decapitated to obtain the brain. Posteriorly, the hippocampus was obtained for quantification of nitrite production, malondialdehyde (MDA), and MDA + 4-hydroxinonenal (4-HDA).

The total protein was quantified by the Sedmak and Grossberg method (1997) using bovine serum albumin (BSA) as standard. Hippocampus was homogenized in PBS 1X solution, in a relation 1 : 4, and the supernatant was pulled apart by a spinning process at 12500 rpm for a period of 30 min at 4°C. Proteins were quantified in 2 *μ*L of supernatant plus 500 *μ*L of Coomassie Brilliant Blue G reactant 0.06% and finally brought to 1 mL with distilled water. The result of the reaction was read in a spectrophotometer (SpectrumVis SP1105) at 620 nm. Protein concentration was determined by interpolation of the optic density of the samples in a BSA standard curve (1 to 10 *μ*g), which was determined parallel in each trial.

The nitric oxide was analyzed through nitrite ion (NO_2_^−^) content in tissue supernatant by the Griess method (Chao y cols., 1992). The Griess reactant was a compound with equal volumes of N-(1-naphthyl)ethylenediamine dihydrochloride 0.1% dissolved in distilled water and sulfanilamide 1.32% (dissolved in acetic acid 60%). The colorimetric reaction was induced by the addition of 100 *μ*L of Griess reactant to 100 *μ*L of supernatant and was brought to 1 mL with distilled water. After 5 minutes of centrifugation at 500 rpm, the reaction proceeds were read in a spectrophotometer (SpectrumVis SP1105) at 540 nm. The NO2 concentration was determined by the interpolation of the optic density of the samples in a NaNO2 standard curve (0.5 to 10 *μ*L), which was parallel determined in each trial.

The determination of MDA and 4-HDA was counted in the samples to analyze the generation of lipid peroxidation products as oxidative stress markers, using the N-methyl-2-phenyl-indole as chromogenic reactant (10.3 mM). 650 *μ*L of solution which consists of N-methyl-2-phenyl-indole dissolved in a mixture of acetonitrile : methanol (3 : 1) was added to 100 *μ*L of supernatant; the solution was vigorously shaken and after that 150 *μ*L of HCL or methanosulfonic acid 35% was added. The reaction was incubated at 45°C for 45 minutes or one hour, respectively, then it was allowed to cool for 5 minutes and finally was centrifuged at 3000 rpm for 15 minutes. Later, the reaction proceed was read in a spectrophotometer (SpectrumVis SP1105) at 586 nm. The MDA and 4-HAD concentration was determined by the interpolation of the optic density of the samples in a 1,1,3,3-tetramethoxypropane standard curve (0.5 to 10 *μ*L), which was determined parallel in each trial.

The dose of 10 mg/kg was chosen for a better antioxidant activity.

### 2.3. Antioxidant Effect of Resveratrol

Four groups of rats were formed: control, vehicle (10% ethanol), vitamin E (2 mg/kg/day), and resveratrol (10 mg/kg/day). The rats were administrated for different periods (2, 4, 6, and 8 months, thus correspond to 5, 7, 9, and 11 months age, resp.). Resveratrol was LEMI & JO Resveratrol® extracted from the root of *Polygonum cuspidatum*; the number of rats in each period was *n* = 16, except for the 8 months administrated, where the number was *n* = 32. It is important to mention that vitamin E was selected to compare the antioxidant effect of resveratrol, because this vitamin is the principal antioxidant against oxidative damage in plasma and erythrocytes [5, 6].

#### 2.3.1. Behavioral Tests

At the end of the corresponding period in each group, the rats were sacrificed, except for the group administered for 8 months, in which before its sacrifice, behavioral tests of the type NOR (novel objects recognition) were realized [18]. The NOR test is suitable for this type of experimental model since it presents advantages over other tests that compromise the integrity of experimental animals, besides that it does not involve positive or negative reinforcements. It is based on spontaneous behavior when accessing a novelty and generating an apparent “unconditioned preference.”

Two previous analyses were performed to obtain the recognition index: the object recognition time was measured and the number of contacts (direct interaction of the animal with the object) was counted. The data from these analyses correspond to the recognition index, so that the NOR test is accurately condensed with this indicator. In order to analyze the data obtained, the recognition index with a value of >0.5 corresponds to a process of consolidation of learning, and on the contrary, an index < 0.5 indicates a lack of interest in recognizing the novel objects.

#### 2.3.2. Effect on the Hippocampus Cytoarchitecture

64 animals were perfused with 1X PBS solution through the left ventricle, cutting the descending aorta artery, and the brain was dissected and preserved in 4% formalin. 30 *μ*m coronal sections in the vibratome were obtained, and for histological studies, the Nissl modified staining was performed [19]. These dyes can bind not only to the DNA content of the cell nuclei but also to the RNA, that is highly concentrated in rough endoplasmic reticulum and ribosomes (Nissl substance) in the cytoplasm. Through the Nissl staining, cell ordering and changes in the cytoarchitecture of the hippocampal CA1 and CA2 structures in the brain tissue sections were observed.

### 2.4. Statistical Analysis

For all the experiments, the data obtained were expressed as a mean ± standard error of the media (SEM). Statistical analysis was developed through ANOVA and the Dunnett posttest.

## 3. Results and Discussion

The present work shows the antioxidative effect of resveratrol in rats at different ages. We determined that 10 mg/kg produces a decrement in oxidative stress of the rats from two months of administration to eight; also, we observed that resveratrol produced an increment in the cognitive process and a major conservation of cytoarchitecture of the CA1 and CA2 of the hippocampus in rats. These animals and the NOR test were employed because they are general models in biomedical research. It has been shown that rodents (mice and rats) are very useful for to obtain adequate and uniform results.

**Figure 1:**
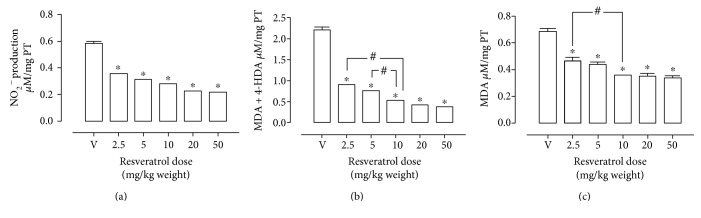
Resveratrol antioxidant effect on the hippocampus: (a) Nitrite levels. Analyzed through Griess method, the nitrite decrease observed is dose-dependent. (b) MDA + 4-HAD levels. It can be observed that resveratrol concentration increment to 10 mg/kg gives the best antioxidant activity. (c) MDA level. The optimum decrease is presented in doses of 10 mg/kg. The lipid peroxidation index was determined by the Gérard-Monnier. The data are means ± standard error of the mean SEM for *n* = 4 rats. ^∗^Significantly different from the vehicle group *p* < 0.001 (one-way ANOVA). ^#^Significantly different from the group 10 mg/kg/weight *p* < 0.05 (ANOVA, Tukey's method).

In the first place, the dose of resveratrol for subsequent experiments was determined.  Figure 1(a) shows that the vehicle group had a concentration of nitrites of 0.6 ± 0.0152 *μ*M/mg of total protein; resveratrol administration during 2 months produced a decrement of 39 ± 1.6%, 48 ± 0.15%, 53 ± 0.39%, and 62 ± 0.54% in the groups administrated with 2.5, 5, 10, and 20 mg/kg of resveratrol, respectively. To determine lipoperoxidation, MDA + 4-HDA (Figure 1(b)) was measured; a decrement of 60 ± 0.06%, 66 ± 0.17%, 76 ± 0.19%, and 81 ± 0.38% in the groups 2.5, 5, 10, and 20 mg/kg of resveratrol was found. On the other hand, individual value of NMDA (Figure 1(c)) showed a similar decrease in the different groups (32 ± 2%, 36 ± 2.3%, 48 ± 0.54%, and 49 ± 2.8%, resp.). Based on these results, it was determined that the appropriate dose that produced antioxidative effect was 10 mg/kg. For analysis, one-way ANOVA and Dunnett's posttest were used. Oral administration of resveratrol promoted a significant decrease in MDA+ 4-HDA levels in the hippocampus. This finding is consistent with previously reported studies in which the appropriate dose was found to be 12.5 mg/kg in subacute administration [20] performed intraperitoneally. In contrast to this trial, the oral administration was the administration of choice and the appropriate dose was 10 mg/kg in weight. It is important to emphasize the importance of this dose, since the administration of higher doses (20 and 50 mg/kg) did not promote a significant increase of the antioxidant activity according to this indicator.

**Figure 2:**
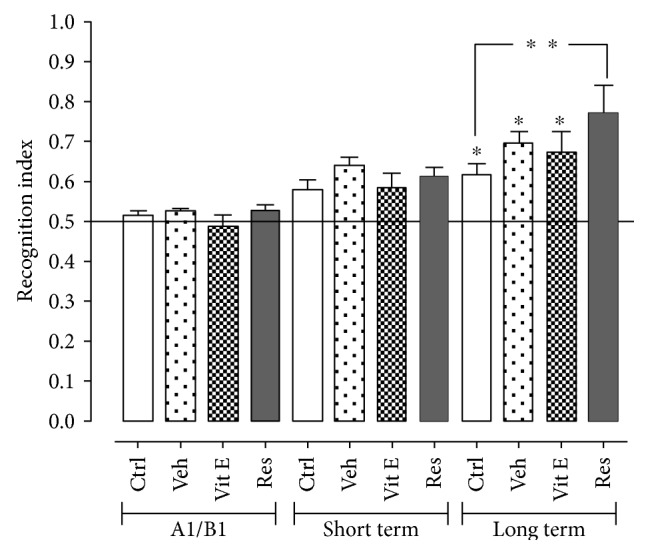
Effect of resveratrol on short-term and long-term memory using the novel object recognition (NOR) test. There are statistically significant differences between the long-term memory of the control compared to the administered group with resveratrol. The data express the mean ± SEM for *n* = 8 rats. ∗ indicates *p* < 0.05 with respect to exposure A1/B1 of its corresponding group. ∗∗ indicates *p* < 0.05 with respect to the control group in long-term memory (ANOVA and Dunnett's test).

The novel object recognition (NOR) test was applied to the 8-month groups to evaluate the possible protective effect of resveratrol on the hippocampus, and the conservation of cognitive abilities and results are shown in Figure 2. According to the behavioral performance evaluation, the recognition index (parameter relating the number of contacts and the time invested in carrying it out) in the control group was 0.6 ± 0.052 in short- and long-term memory 0.061 ± 0.028. As for the group administered with vehicle, an index of 0.67 ± 0.02 and 0.69 ± 0.027 in the short- and long-term memory, respectively, was reported. The index in the group administered with vitamin E in short- and long-term memory was 0.58 ± 0.037 and 0.67 ± 0.052. Finally, in the group administered with resveratrol, it presented an index of 0.61 ± 0.037 in the short-term memory and 0.77 ± 0.115 in the long-term memory, which represented a 20% increase in comparison with the recognition index observed in the control group. There were no statistically significant differences in other groups.

For the NOR trial, all groups present in this evaluation presented the consolidation of the learning process, including the control group. Additionally, it is observed that the group administered with vehicle has an index similar to that shown by the controls, which allows to rule out that there is any additional effect on the evaluated animals when administering 10% ethanol. On short-term memory, there were no statistically significant differences with their controls or between groups, which reflects that the animals did not recognize a great extension of the novel objects that were exposed to them and their exploration was not significant. As for the group administered with resveratrol, a significant increase in the index of recognition in the long-term memory was found. This important change may be due to the fact that long-term memory is a type of declarative memory whose development involves the cortical area and the parahippocampal gyrus, where associations and conjunctions among stimuli are carried out, that is, the neurochemistry of the hippocampus is essential for recognition memory [Stanley et al., 2012], so it indicates that long-term memory is being favored by the protective effect of resveratrol on this region. It has been reported that this neuroprotective effect is due to the preservation of hippocampal integrity, and the treatment selectively protects neurons in the CA1 and CA2 regions [21].

**Figure 3:**
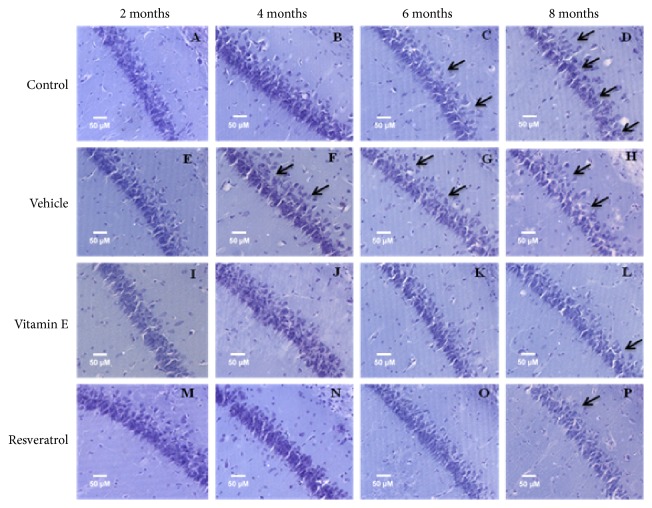
Effect of resveratrol on the hippocampus cytoarchitecture in the CA1 region in 2, 4, 6, and 8-month treatment rats (20×). (a) Control group of 2 months. (b) Control group of 4 months. (c) Control group of 6 months. (d) Control group of 8 months. The CA1 region of the hippocampus and the arrangement of the cells are observed; the cytoarchitecture begins to be modified with respect to the increase of the age. (e) Vehicle group of 2 months. (f) Vehicle group of 4 months. (g) Vehicle group of 6 months. (h) Vehicle group of 8 months. In these groups, greater dispersion of the cells is observed. (i) Group administered with vitamin E for 2 months. (j) Vitamin E group of 4 months. (k) Vitamin E group of 6 months. (l) Vitamin E group of 8 months. During the different periods, it is observed that the CA1 region is conserved; however, the space that is observed between the cells is remarkable. (m) Group administered with resveratrol for 2 months. (n) Group administered with resveratrol for 4 months. (o) Resveratrol group of 6 months. (p) Resveratrol group of 8 months. The structure of CA1 is shown with an order in the arrangement of the cells and the axonal projections are observed aligned, the integrity of the structure is conserved largely and the scattered cells are scarce, and even at 8 months, the resveratrol group is observed with less cellular disorganization. The arrows indicate areas where cells are scattered in the CA1 region.

**Figure 4:**
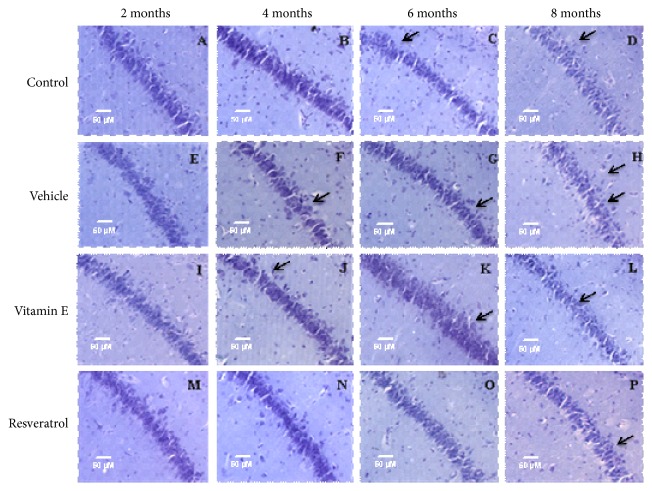
Effect of resveratrol on the hippocampus cytoarchitecture in the CA2 region in rats of 2, 4, 6, and 8 months of treatment (20×). (a) Control group of 2 months. (b) Control group of 4 months. (c) Control group of 6 months. (d) Control group of 8 months. The CA2 region of the hippocampus is observed, and it is interesting to note the arrangement of the cells and the axonal projections; this region does not result in severe alterations as observed, but the gradual changes lead to the disorganization that as observed in the CA1 region is present in different brain regions. (e) Vehicle group of 2 months. (f) Vehicle group of 4 months. (g) Vehicle group of 6 months. (h) Vehicle group of 8 months. Cellular cytoarchitecture is compromised over time, and changes due to cellular disorganization are more noticeable. (i) Group administered with vitamin E for 2 months. (j) Vitamin E group of 4 months. (k) Vitamin E group of 6 months. (l) Vitamin E group of 8 months. The organization of cells is conserved and changes with respect to time are minimal. (m) Group administered with resveratrol for 2 months. (n) Group administered with resveratrol for 4 months. (o) Resveratrol group of 6 months. (p) Resveratrol group of 8 months. The CA2 region of the hippocampus retains its integrity until after 6 months of treatment and then presents characteristic changes of aging. The arrows indicate areas where there are scattered cells or changes in the organization of cells.

CA1 (Figure 3()) and CA2 (Figure 4()) regions of the hippocampus were visualized, which are closely related to memory and learning processes. The changes in the cytoarchitecture refer especially to the cellular organization, where the arrangement and order of the axonal projections are observed. In the hippocampal CA1 region, we observed a gradual change in the cytoarchitecture due to the time of administration and the type of treatment. In the intact specimens (Figures 3()(a), 3()(b), 3()(c), and 3()(d)), a subtle modification of the cellular confluence is seen from 2 to 8 months of treatment. In addition, it shows a greater dispersion in the cells and even a decrease in the number of cells. On the other hand, in the groups administered with vehicle (Figures 3()(e), 3()(f), 3()(g), and 3()(h)), the changes are evidenced due to the time. The cellular disorganization is appreciable from the beginning of the administration and the changes are drastic when the group of 2 months is compared with the last period of treatment. In contrast to these observations, the groups administered with antioxidants (Figures 3()(i), 3()(j), 3()(k), 3()(l), 3()(m), 3()(n), 3()(o), and 3()(p)), that is, with vitamin E and resveratrol, show that the integrity of the structure is better preserved, especially when administering resveratrol (Figures 3()(m), 3()(n), 3()(o), and 3()(p)), where the axonal projections are observed to be aligned and ordered, and conserve a greater number of cells in comparison with the other groups.

The analysis and visualization of the CA2 region of the hippocampus revealed subtle changes that were reproduced throughout the treatment time. The 2-month control group (Figure 4()(a)) is observed with a greater confluence of cells, which through the months, is diminished until there is an evident dispersion in the arrangement of the cells in the last group of 8 months (Figure 4()(d)). The cellular disorganization that can be distinguished in the group administered with vehicle is evident in relation to the chronology of the treatment, where in addition a reduction in the number of cells in this region becomes appreciable, and the dense layer of cells is diminished, being observed spaces between cells. In the groups administered with vitamin E (Figures 4()(i), 4()(j), 4()(k), and 4()(l)), the CA2 region is observed with better conservation and cellular order and is appreciable that it is considerably similar to the control group; however, after 8 months of treatment, it is poorly defined and with a decrease in cell density (Figure 4()(l)). In the groups treated with resveratrol (Figures 4()(m), 4()(n), 4()(o), and 4()(p)), the integrity of the CA2 region is conserved until after 6 months of treatment (Figure 4()(o)), and after 8 months, the cytoarchitecture was modified (Figure 4()(p)), since an undefined region is observed and with very dispersed cells in its conformation.

**Figure 5:**
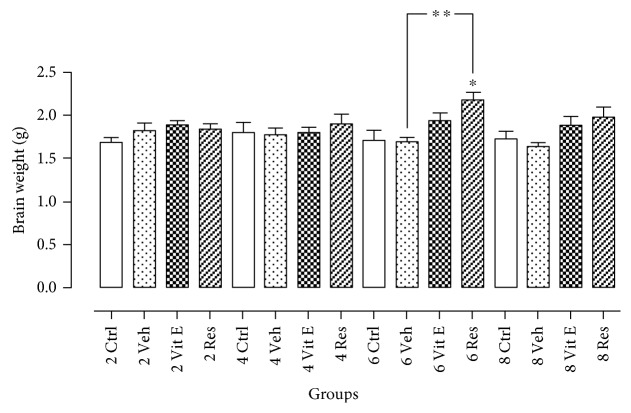
Effect of resveratrol on brain atrophy. Reduced brain size is a hallmark of aging. Comparing each of the groups, the resveratrol group has a tendency to avoid the decline in brain mass. Statistically significant differences were observed between the brain weights of animals treated with resveratrol for six months compared to control group and those administered with vehicle. The data express the mean ± SEM for *n* = 4 rats, with a *p* < 0.05. ∗ indicates *p* < 0.05 with respect to the 6-month control group. ∗∗ indicates *p* < 0.05 with respect to the 6-month vehicle group (ANOVA and Dunnett's test).

In addition to these observations, it was considered to compare brain mass between the different treated groups, because a decrease in brain mass is related to the process of cerebral atrophy, which is another characteristic of the aging process. The differences in the brain mass were analyzed (Figure 5()), and these differences were reported between the brain weights of animals administered with resveratrol compared to those treated with vehicle for six months. Although no statistical differences were found in other groups, it is important to note that in all groups administered with a vehicle, there is a trend of brain mass decreasing and, also, a lesser extent in the control groups. This evidence might suggest that if experimental animals were brought to a greater age, significant changes would be reflected in the difference in brain mass. It has been reported that major changes are evident in rats during adulthood, specifically in older adults. In correspondence to the lifetime and the weight of the rat, the animals that were intervened in this experimentation were in the young adult to the middle-aged adult phase of life. To reach the aging stage in the rat model, age ranges from 15 to 24 months according to the correlation made for this model organism [22, 23]; therefore, the most severe changes are significant and forceful until reaching this age.

With this evidence, it can be suggested that the efficiency of the antioxidant activity of resveratrol in rats was transcendental practically throughout the period between 2 and 8 months of administration, the cerebral integrity and the cognitive functions seem to be conserved when chronic resveratrol is consumed, the oral administration of resveratrol for 8 months allowed a development in cognitive performance, particularly on long-term memory, and cellular disorganization in the CA1 and CA2 region of the hippocampus was attenuated with chronic resveratrol administration.
