# EXPRESSION OF CONCERN: Effect of Chronic Administration of Resveratrol on Cognitive Performance during Aging Process in Rats

**DOI:** 10.1155/omcl/9807402

**Published:** 2026-03-25

**Authors:** Oxidative Medicine and Cellular Longevity

**EXPRESSION OF CONCERN:** A. R. Navarro‐Cruz, R. Ramírez y Ayala, C. Ochoa‐Velasco, E. Brambila, R. Avila‐Sosa, S. Pérez‐Fernández, J. C. Morales‐Medina, and P. Aguilar‐Alonso, “Effect of Chronic Administration of Resveratrol on Cognitive Performance during Aging Process in Rats,” *Oxidative Medicine and Cellular Longevity* 2017, no. 1 (2017): 8510761, https://doi.org/10.1155/2017/8510761.

This Expression of Concern for the above article, published online on 15 October 2017 in Wiley Online Library (wileyonlinelibrary.com), has been published by agreement between the journal Editor‐in‐Chief, Jeannette M Vasquez Vivar; and John Wiley & Sons Ltd. The Expression of Concern has been agreed because of figure concerns that were raised by *Actinopolyspora biskrensis* via PubPeer [1]. Specifically, panel “Resveratrol – 8 months” in Figure 3 shares unexpected similarities with panel, “Control – 8 months” in Figure 4.

The authors stated that this was due to an honest error, but the original photographs were lost after the passing of the corresponding author. The Editor believes that the paper appears otherwise reliable and, as a result, the decision to issue an Expression of Concern was made to alert and inform readers.

## References

[1] Actinopolyspora biskrensis , Effect of Chronic Administration of Resveratrol on Cognitive Performance during Aging Process in Rats, *PubPeer* (2022), https://pubpeer.com/publications/7F8C4674386F09C069AADFFF05714C.

